# Supplementary material for: Mechanism of polyadenylation-independent RNA polymerase II termination
Source: Nat Struct Mol Biol. 2024 Oct 18;32(2):339–45. doi: 10.1038/s41594-024-01409-0 (PMC11832416; doi:10.1038/s41594-024-01409-0)

nature structural & molecular biology

Supplementary information

https://doi.org/10.1038/s41594-024-01409-0

Mechanism of polyadenylation-independent RNA polymerase II termination

In the format provided by the authors and unedited

SUPPLEMENATARY FIGURES

Supplementary Figure 1 | *In vitro* transcription termination assay

Schematic cartoon representation of the *in vitro* transcription termination assay performed to test the function of our minimal pre-TC. The colour codes are consistent with Fig. 1 and the components of the assay are labelled accordingly.

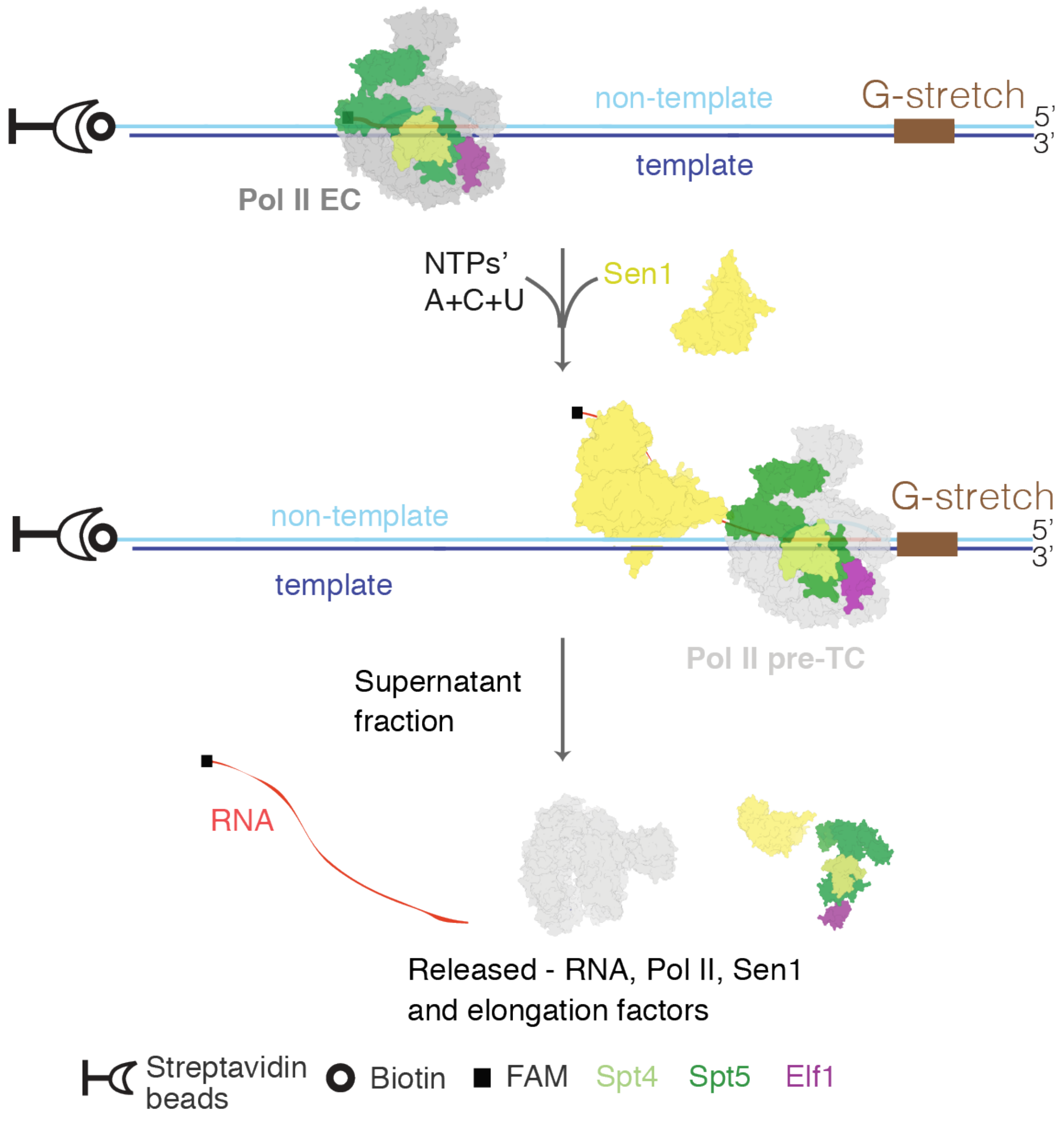

Supplementary Figure 2| 3D FSC plots of cryo-EM maps

a-d: Directional 3D FSC of all four pre-TC maps, determined using 3DFSC server[21].

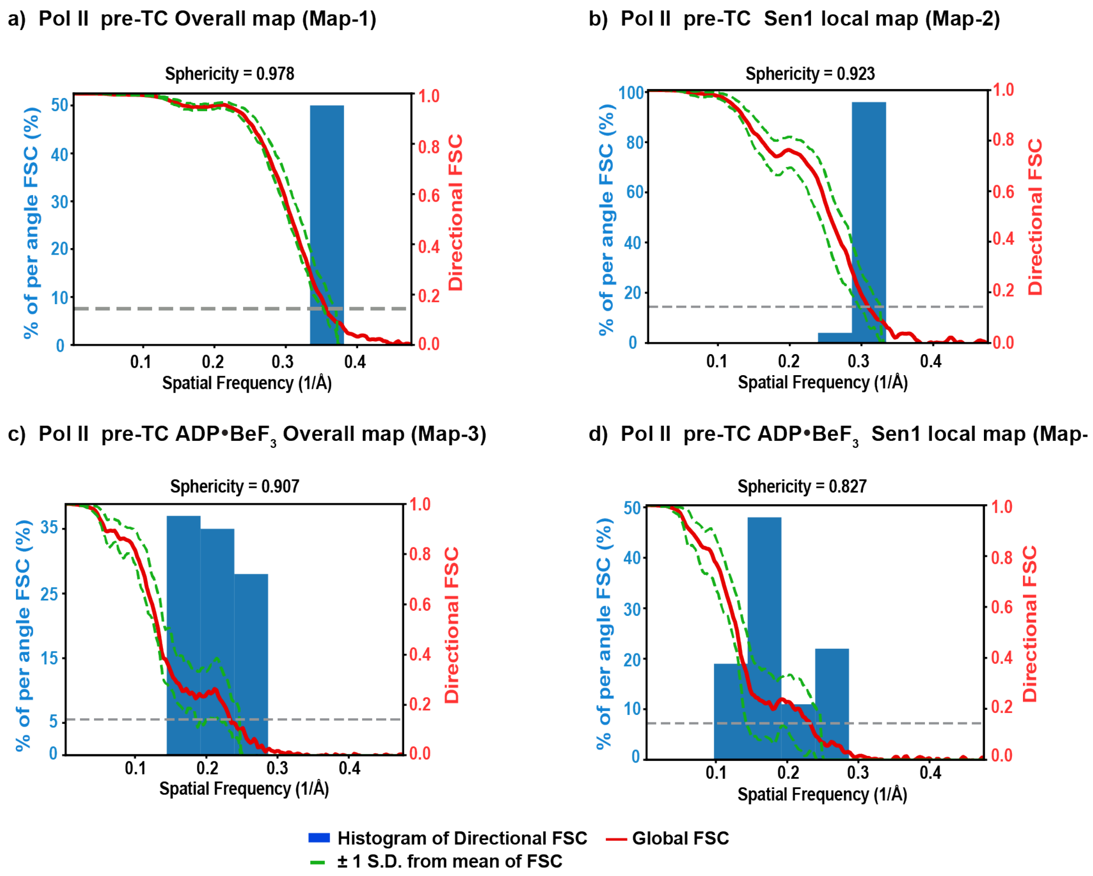

Supplementary Figure 3 | Pol II active site in the pre-TC

Comparison of the DNA:RNA hybrid between the yeast Pol II pre-TC in a fully translocated state and human Pol II PEC in a tilted state (PDB code: 6GML). Nucleotides base-paired at the -1 and +1 positions are highlighted with dotted (yeast pre-TC) and solid (human PEC) lines. Though the same scaffold shows a tilted DNA-RNA hybrid at the active site of promoter-proximally paused human Pol II, its absence in our structure could be due to the *in vitro* reconstitution of the Pol II pre-TC or due to the lack of the NELF complex [8],which is not conserved in yeast.

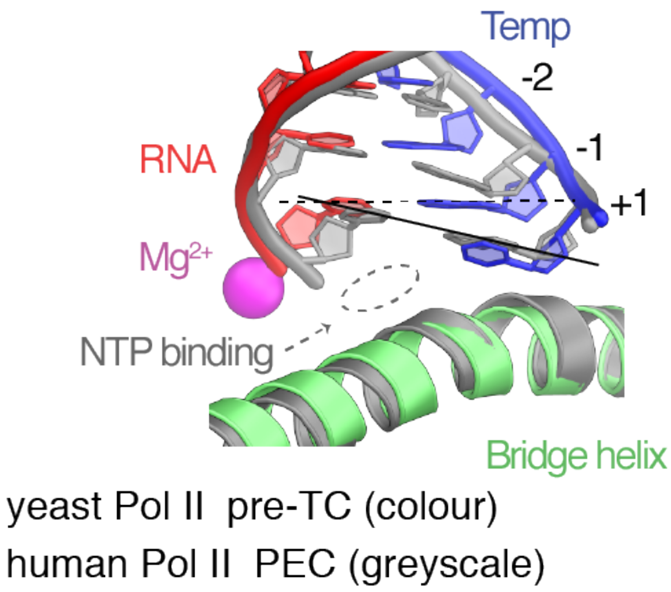

Supplementary Figure 4 | Sequence alignment of Sen1 and Rpb3 interaction regions. Sequence alignments were performed with the regions of individual subunits for which the structure has been determined in this study. T-Coffee algorithm[22] was adopted to obtain a structure based sequence alignment which was then visualized using ESPript[23]. Residues with identity above 70% are coloured: red (identical) and yellow (homologous). The residues involved in inter-subunit interactions between Rpb3 and Sen1 (Fig. 2a) are highlighted with stars; colour codes are consistent with Fig. 2.

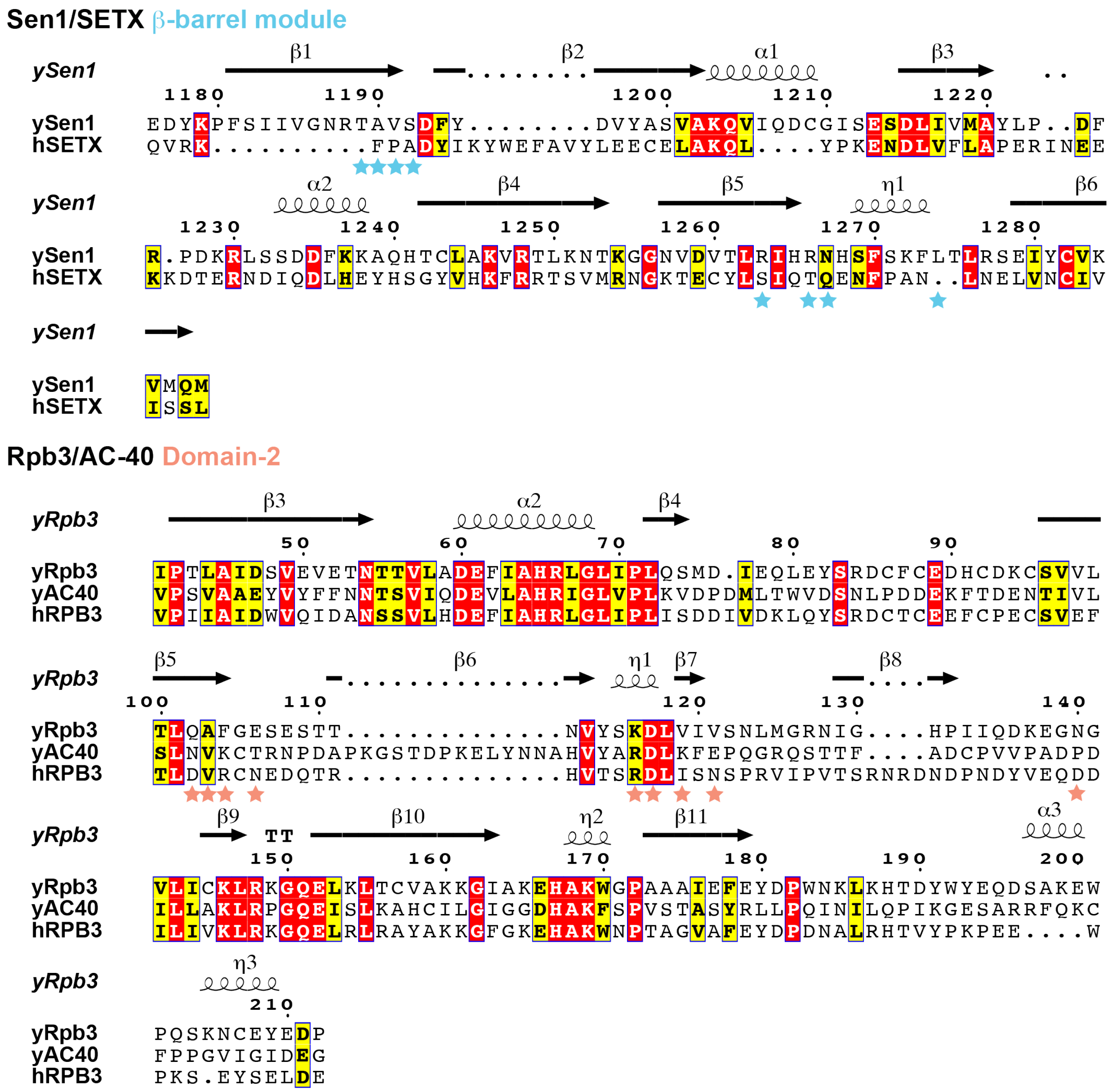

Supplement: Supplementary file 1 — Supplementary Figs. 1–4. [file 41594_2024_1409_MOESM1_ESM.pdf]
