# Supplementary material for: Mechanism of polyadenylation-independent RNA polymerase II termination
Source: Nat Struct Mol Biol. 2024 Oct 18;32(2):339–45. doi: 10.1038/s41594-024-01409-0 (PMC11832416; doi:10.1038/s41594-024-01409-0)
Supplement: Supplementary file 6 — Unedited raw images of all the gels used. All three replicates of gels for Extended Data Fig. 10b are also furnished. [file 41594_2024_1409_MOESM6_ESM.pdf]

Extended Data Figure 10a

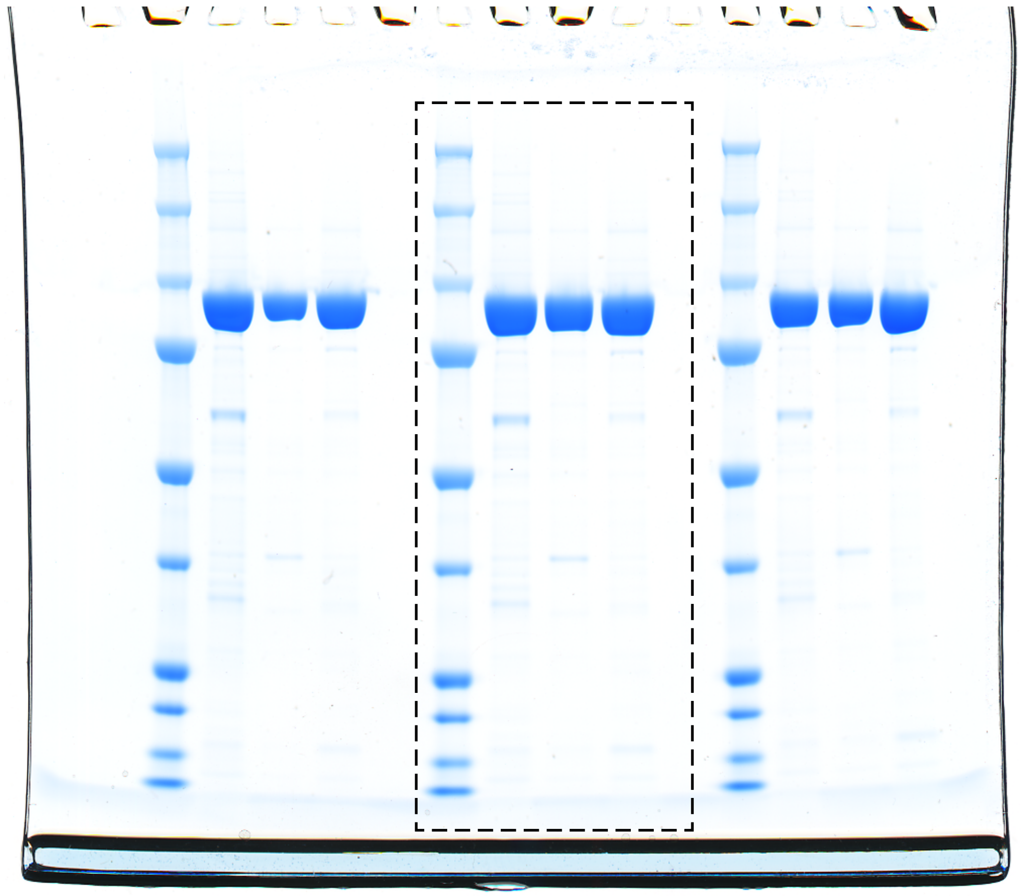

Extended Data Figure 10b

Replicate-1

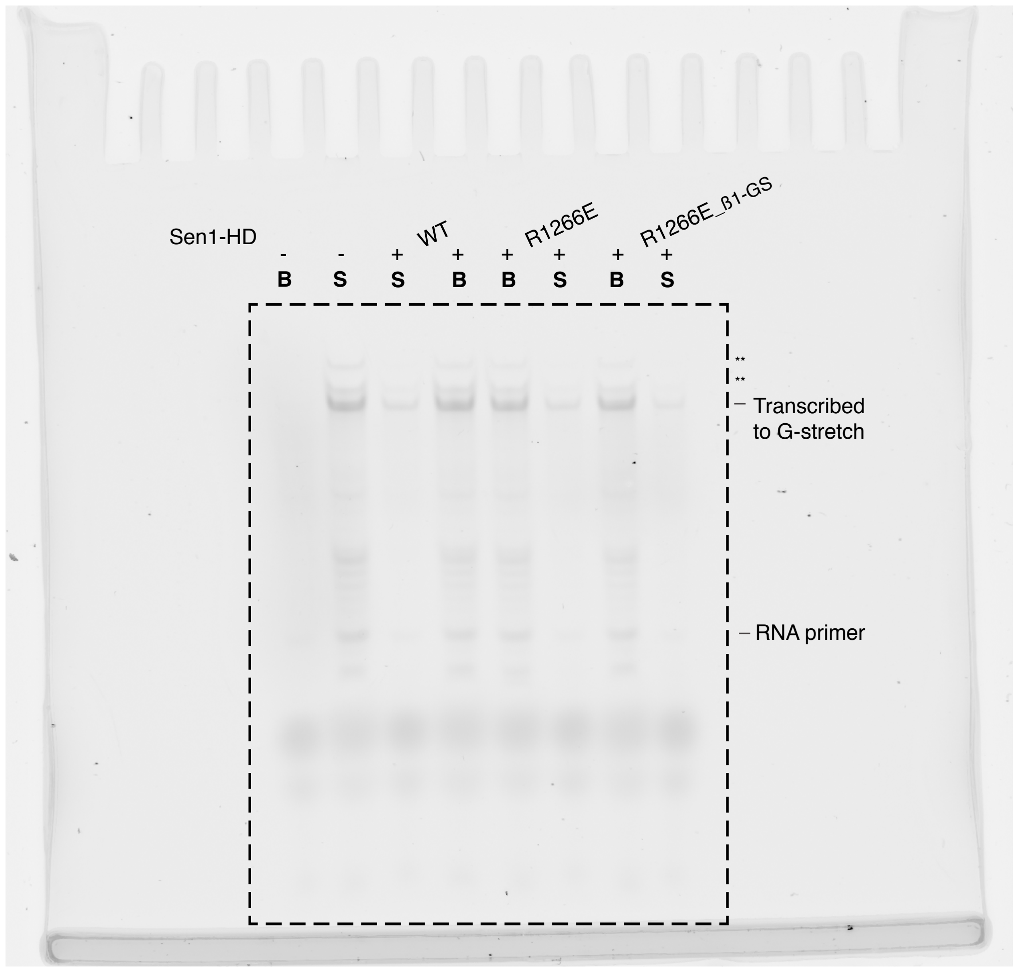

Replicate-2

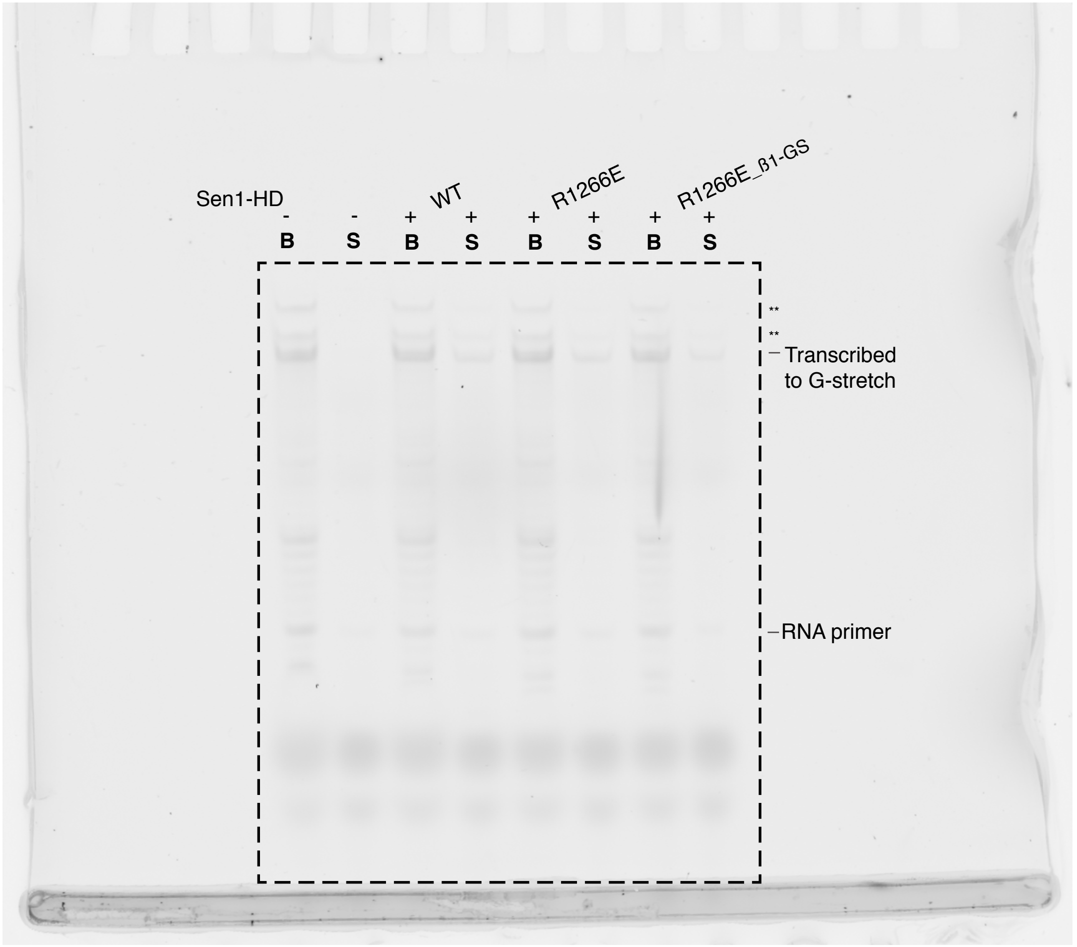

Replicate-3

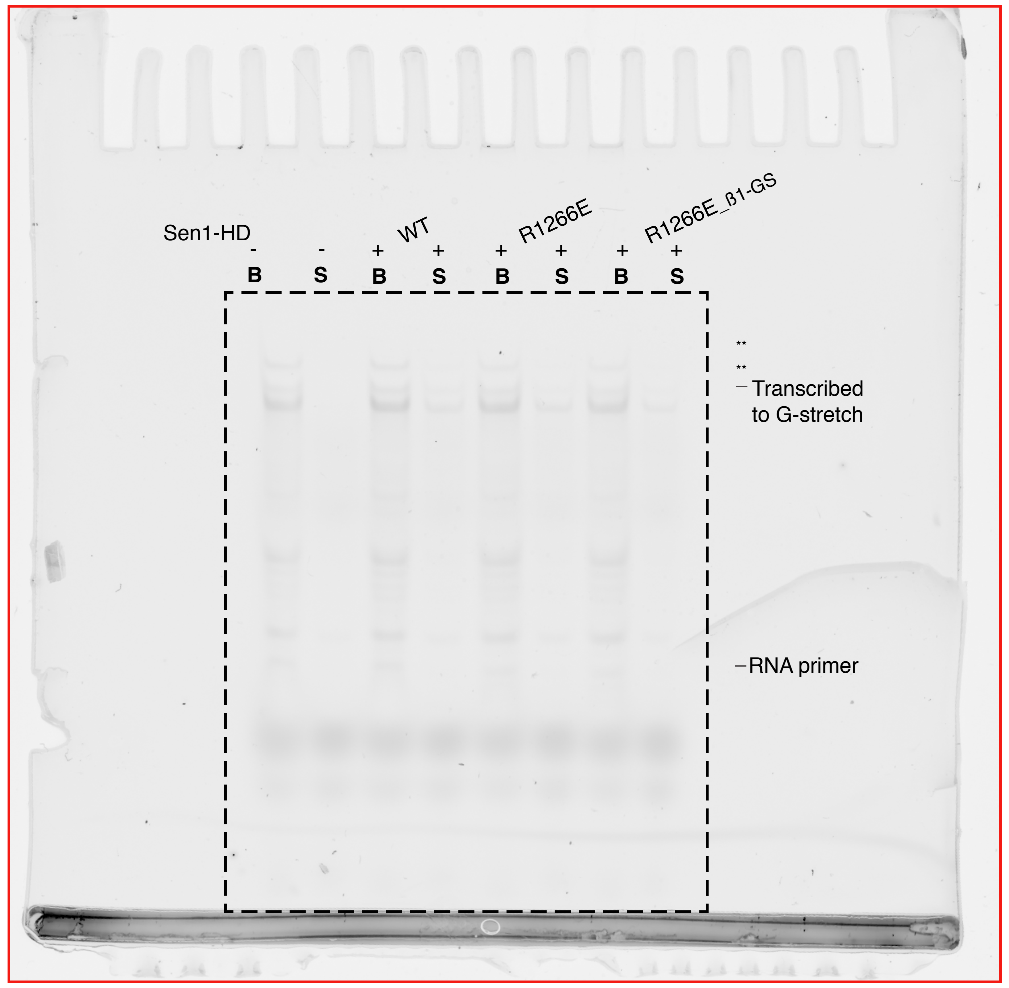

Figures: Source data of SDS-PAGE gel presented in Extended Data Figure 10a, and Urea-PAGE gels in1c. Black boxes represent the lane used in the in the respective figure. Replicate-3 used in Extended Data Figure 10b is highlighted with a red outline.
